# Clinical Trial Diversity in Oncology: FDA Takes Action with Post–Marketing Requirements or Commitments

**DOI:** 10.1093/oncolo/oyac228

**Published:** 2022-11-01

**Authors:** Janice Kim, Robert Kester, Gideon Blumenthal

**Affiliations:** Merck & Co., Inc., Kenilworth, NJ, USA; Merck & Co., Inc., Kenilworth, NJ, USA; Merck & Co., Inc., Kenilworth, NJ, USA

## Abstract

In recent years, there has been a renewed focus on promoting the inclusion of patients from racial and ethnic minority groups in oncology clinical trials. FDA Oncology has long pointed to the underrepresentation of racial minorities in registration trials leading to approval. US FDA’s Guidance on diversity discusses how diversity could be handled within clinical trials, giving recommendations on broadening eligibility criteria, inclusive trial practices, and alternative trial designs. While there is no specific guidance from the FDA on cancer clinical trials, the recommendation is to include a representative population applicable to the US population. With the recent renewed focus on diversity in oncology clinical trials, FDA Oncology has recently asked for the completion of a Diversity Plan during drug development and has issued post–marketing commitments and requirements at the time of approval. As FDA has started to issue post–marketing requirements or commitments regarding diversity in 2020, we sought to analyze the post–marketing studies asking for a study of racial and ethnic minorities issued by the FDA’s Office of Oncologic Diseases (OOD). The analysis demonstrated the need to increase the enrollment of a diverse patient population in cancer clinical trials.

## Teaser

There is renewed focus on promoting inclusion of patients from racial and ethnic minority groups in oncology clinical trials. This commentary analyzes postmarketing studies racial and ethnic minorities issued by the FDA’s Office of Oncologic Diseases.

## Background

In recent years, there has been a renewed focus on promoting the inclusion of patients from racial and ethnic minority groups in oncology clinical trials. In 2020, the US FDA finalized a Guidance on “Enhancing the Diversity of Clinical Trial Populations” which recommends that Sponsors include a plan for the inclusion of clinically relevant populations no later than the end of the phase II meeting. Most recently the US FDA issued a draft Guidance on “Diversity Plans to Improve Enrollment of Participants from Underrepresented Racial and Ethnic Populations in Clinical Trials” which describes in further detail a diversity plan to be submitted by Sponsors addressing how they plan to enroll representative numbers of patients from underrepresented racial and ethnic populations.^1^ FDA Oncology has long pointed to the ­underrepresentation of racial minorities in registration trials leading to approval.^2,3^ In addition to the FDA, several key oncology stakeholders have recognized the need for more inclusion of underrepresented minorities in cancer clinical trials.^4^ In addition, 21 CFR 314 outlines that applications based solely on foreign clinical data may be approved if the data are applicable to the US population and US medical practice.^5^ Given the relatively lower rates of US accrual in some registration studies, the issue of underrepresentation of minority patients may be exacerbated.

US FDA’s Guidance on diversity discusses how diversity could be handled within clinical trials, giving recommendations on broadening eligibility criteria, inclusive trial practices, and alternative trial designs.^6^ Broadening eligibility ­criteria were likely added as a recommendation because eligibility criteria in a cancer clinical trial can be narrow and strict, ­making it difficult for ethnic and minority patients to enroll in a trial due to known co-existing co-morbidities that might exclude them from the trial. For example, it is widely known that type II diabetes is more prevalent in the Black and Native American populations, which may make them more likely to have impaired kidney function as a result. In addition, some cancer clinical trials do not account for differences in racial and ethnic laboratory value differences when determining the eligibility criteria, which could preclude certain subpopulations from enrolling in a clinical trial.

While there is no specific guidance from the FDA on cancer clinical trials, the recommendation is to include a representative population applicable to the US population. In addition, ICH E17 stresses that a well-conducted Multi-Regional Clinical Trial (MRCT) would have input from regulatory authorities, would permit analyses of regional consistency, and would potentially address concerns regarding applicability to the US patient population, including underrepresented minorities.^7^ In a recent perspective piece in Lancet Oncology, FDA Oncology leadership stressed that MRCTs are the preferred means of drug registration and that the greater diversity in MRCTs might provide additional information to assist the United States in addressing the underrepresentation of racial and ethnic minorities in drug development.^8^

With the recent renewed focus on diversity in oncology clinical trials, FDA Oncology has recently asked for the completion of a Diversity Plan during drug development and has issued post–marketing commitments and requirements (authority to require sponsors to conduct PMR/PMCs was granted to the FDA under the 2007 FDA Amendments Acts (FDAAA)) at the time of approval.^9^ As FDA has started to issue post–marketing requirements or commitments that ­included language on diversity in 2020, we sought to analyze the post–marketing studies asking for a study of racial and ethnic minorities issued by the FDA.

## Methods

Two years’ worth of oncology drug approvals were examined between January 2020 and December 2021 within the Division of Oncology 1 (focusing on women’s and genitourinary cancers), Division of Oncology 2 (focusing on thoracic, head and neck, rare, and pediatric cancer), Division of Oncology 3 (focusing on melanoma, sarcoma, and gastrointestinal cancer), Division of Hematologic Malignancies 1 (focusing on leukemia), and Division of Hematology Malignancies 2 (focusing on lymphoma and multiple myeloma) as 2020 was the first year a PMR or PMC that included language on diversity was issued by FDA’s Oncology Office. For each drug approval, the FDA approval letter was examined to determine if a post–marketing commitment (PMC) or post–marketing requirement (PMR) to conduct or report on data among US racial and ethnic minority patients was included in the approval letter. After identifying the PMC or PMR, the wording was analyzed. In addition, for each application that had a PMR or PMC issued, Drugs@FDA was searched to determine if the FDA Summary Basis of Approval (SBoA) were available.^10^ If FDA SBoA were available any additional information regarding the PMR or PMC that was issued was analyzed.

## Results

**Figure 1.**
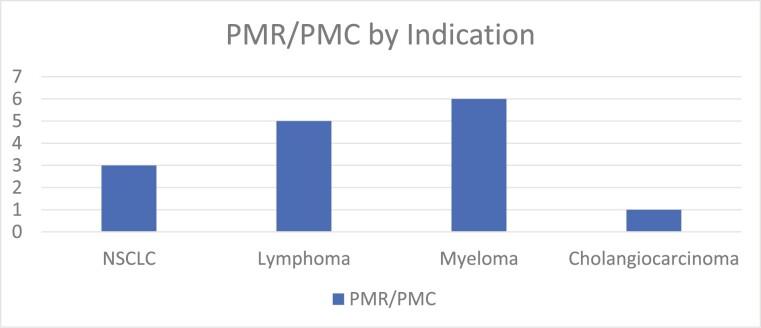
FDA PMR or PMC issued by oncologic indication.

Among 123 oncology drug approvals, 15 PMRs or PMCs related to diversity were identified in FDA approval letters and assessed. The first PMR or PMC to be issued was for Diffuse large B-cell lymphoma on July 31, 2020 for ­tafasitamab-cxix. The most recent PMR or PMC to be issued was on November 30, 2021 for daratumumab and hyaluronidase-fihj indicated for multiple myeloma. Multiple myeloma indications had the most diversity PMR or PMC issued, followed by lymphoma, non–small cell lung cancer and cholangiocarcinoma (Fig. 1).

Among the diversity, PMRs and PMCs issued 9 were New Molecular Entity Drug applications and 6 were Supplemental drug applications, 6 were approved under FDA’s traditional approval pathway and 9 were approved under the accelerated approval pathway.

In FDA approval letters, most of the diversity PMRs issued stated to conduct a study and those that did not stated to include an assessment of ongoing studies. PMRs were written such that the Sponsor would have to provide both safety and efficacy data in racial and ethnic minority patients to be more reflective of the US population (Table 1).

**Table 1.** Diversity PMR or PMC issued by FDA in oncology between January 2020 and December 2021.

Generic name	Approval date	Indication	Accelerated or traditional	PMR or PMC	Diversity PMR or PMC wording
Tafasitamab	7/31/20	Diffuse large B-cell lymphoma	Accelerated	PMR	Submit the final report and datasets from a randomized, phase III clinical trial to verify the clinical benefit of tafasitamab in patients with diffuse large B-cell lymphoma. The trial should include sufficient numbers of racial and ethnic minority patients to better reflect the US patient population and allow for the interpretation of the results in these patient populations. Patients should be randomized to receive immunotherapy and/or chemotherapy with or without tafasitamab and lenalidomide. The primary endpoint should be progression-free survival, with secondary endpoints that include overall survival and objective response rate.
Umbralisib	2/5/2021	Marginal zone lymphoma and follicular lymphoma	Accelerated	PMR	Conduct a randomized, phase III clinical trial that verifies and describes the clinical benefit of umbralisib in patients with relapsed or refractory follicular lymphoma and marginal zone lymphoma. The trial should include sufficient numbers of racial and ethnic minority patients to better reflect the US patient population and allow for interpretation of the results in these patient populations. Patients should be randomized to receive immunotherapy with or without umbralisib. The primary endpoint should be progression-free survival, with secondary endpoints that include overall survival and objective response rate
Melphalan ­flufenamide	2/26/2021	Multiple myeloma	Accelerated	PMR	Submit an integrated final report containing data from clinical trials including trial OP-108 to further characterize the exposure of melphalan flufenamide, the increased risk of serious adverse events including hematologic toxicities, and efficacy among US racial and ethnic minorities including Black patients with relapsed or refractory multiple myeloma. Provide the pharmacokinetic analysis in the interim report.
Idecabtagene ­vicleucel	3/26/2021	Multiple myeloma	Regular	PMC	Celgene commits to submit an integrated final report containing data from clinical trials MM-002 and MM-003 to further characterize the safety and efficacy of idecabtagene vicleucel among African-Americans/Blacks with multiple myeloma. The primary objective of this analysis is to evaluate the efficacy of idecabtagene vicleucel in the subpopulation of African-Americans/Blacks with multiple myeloma compared to the subpopulation of Whites, and the secondary objective is safety. Ensure that the representation of the African American subpopulation in the studies is reflective of the Black population in the geographical location/country. Therefore, approximately 15% of the population that is enrolled from the US should comprise of African Americans. Prespecify an analysis plan for safety and efficacy with a justification/rationale of prespecified assumptions for efficacy outcomes.
Isatuximab-irfc	3/31/2021	Multiple myeloma	Regular	PMC	Submit a final report containing data from clinical trials, post–marketing reports, compassionate use/expanded access programs, real–world evidence, and other sources to further characterize the safety and efficacy of isatuximab in combination with carfilzomib and dexamethasone (Isa-Kd) among US racial and ethnic minority patients with multiple myeloma.
Loncastuxumab tesirine-lpyl	4/23/2021	B-cell lymphoma	Accelerated	PMR	Submit an integrated final report containing data from clinical trials to further characterize the exposure of loncastuximab tesirine-lpyl monotherapy and in combination with immunochemotherapy, the increased risk of severe and serious adverse events, including severe neutropenia, and efficacy among US racial and ethnic minority patients with large B-cell lymphoma. Provide the population pharmacokinetic and exposure-response analyses for both efficacy and safety in the interim report.
Amivantamab-vmjw	5/21/2021	Non-small cell lung cancer	Accelerated	PMC	Submit a final report containing data from clinical trials enrolling a sufficient representation of Black or African American patients that is reflective of the US population of patients with EGFR exon 20 insertion mutated NSCLC to further characterize the safety and efficacy of amivantamab-vmjw in Black or African American patients with EGFR exon 20 insertion mutated NSCLC.
Sotorasib	5/28/2021	Non–small cell lung cancer	Accelerated	PMC	Submit a final report containing data from clinical trials enrolling a sufficient representation of African American patients that is reflective of the US population of patients with KRAS G12C mutated non-small cell lung cancer to further characterize the safety and efficacy of sotorasib in African American patients with KRAS G12C mutated non-small cell lung cancer.
Infigratinib	5/28/2021	Cholangiocarcinoma	Accelerated	PMR	Conduct a clinical trial to further characterize the serious adverse reactions of hyperphosphatemia and eye disorders in patients with firstline or refractory cholangiocarcinoma harboring an FGFR2 fusion or other rearrangement receiving alternate dosage(s) regimens of infigratinib. Characterize all serious adverse events including hyperphosphatemia and eye disorders, dose reductions, interruptions, and discontinuations due to serious adverse events. Compare clinical efficacy and safety descriptively across concurrently enrolled, parallel cohorts evaluating the approved infigratinib dosage and an alternate dosage regimen. Include sparse PK samples for exposure–response analyses for efficacy and safety and conduct exploratory PK/PD analysis using serum phosphate levels. Ensure that racial and ethnic minority subjects are adequately represented in the trial population, at a minimum, proportional to the prevalence of FGFR2 alterations in these subgroups in the US population.
Daratumumab and hyaluronidase-fihj	7/9/2021	Multiple myeloma	Regular	PMR	Conduct a clinical study to further characterize the exposure of daratumumab (D) subcutaneous (SC), the increased risk of severe and serious adverse events, including severe neutropenia, and efficacy among US racial and ethnic minority patients with relapsed or refractory multiple myeloma. Include an assessment of the PK, PD, safety, and efficacy of daratumumab SC in combination with other agents including pomalidomide and dexamethasone (Pd) in US racial and ethnic minority patients including Black and Asian patients with relapsed or refractory multiple myeloma in the final study report. The population pharmacokinetic and exposure–response analyses for both efficacy and safety should be updated.
Daratumumab and hyaluronidase-fihj	7/9/2021	Multiple myeloma	Regular	PMC	Submit a final report containing data from clinical trials, post–marketing reports, compassionate use/expanded access programs, real–world evidence, and other sources to further characterize the safety and efficacy of daratumumab (SC) in combination with pomalidomide and dexamethasone among US racial and ethnic minority patients with multiple myeloma.
Zanubrutinib	8/31/2021	Waldenstrom’s macroglobulinemia	Regular	PMC	Conduct a study to further characterize the clinical benefit and safety of zanubrutinib for the treatment of patients with newly diagnosed Waldenström’s Macroglobulinemia with MYD88 mutation. This should include an assessment of the CXCR4 mutation status. In addition, the study should include a sufficient number of patients enrolled in the United States and sufficient numbers of racial and ethnic minority patients to allow for the interpretation of the results in these patient populations. 2. Conduct a study to further characterize the clinical benefit and safety of zanubrutinib in patients with newly diagnosed and relapsed/refractory Waldenström’s macroglobulinemia with MYD88wt. This study should include a sufficient number of patients enrolled in the United States and sufficient numbers of racial and ethnic minority patients to allow for the interpretation of the results in these patient populations. 3. Conduct an integrated analysis containing data from clinical trials and other data sources such as post–marketing reports, real–world evidence and other sources to further characterize the safety and efficacy of zanubrutinib in racial and ethnic minorities with Waldenström’s macroglobulinemia.
Zanubrutinib	9/14/2021	Marginal zone lymphoma	Accelerated	PMR	Conduct a randomized clinical trial that verifies and describes the clinical benefit of zanubrutinib in patients with relapsed or refractory marginal zone lymphoma. The trial should include sufficient representation of racial and ethnic minorities to better reflect the US patient population and allow for interpretation of the results in these patient populations. The primary endpoint should be progression-free survival, with secondary endpoints that include objective response rate and overall survival.
Mobocertinib	9/15/2021	Non–small cell lung cancer	Accelerated	PMC	Conduct an analysis containing data from clinical trials enrolling a sufficient representation of US racial and ethnic minorities, including Black or African American patients, that is reflective of the US population of patients with EGFR exon 20 insertion–mutated NSCLC
Daratumumab and hyaluronidase-fihj	11/30/2021	Multiple myeloma	Regular	PMC	Conduct an integrated study analysis containing data from clinical trials, post–marketing reports, compassionate use/expanded access programs, real–world evidence, and other sources to further characterize the safety and efficacy of daratumumab (SC) in combination with carfilzomib and dexamethasone among US racial and ethnic minority patients with multiple myeloma.

In available review packages on Drugs@FDA, FDA comments emphasized concerns regarding data generalizability if underrepresented populations were not included in the study within the indicated population. Within the FDA approvals that had diversity PMR or PMC issued, minority patients ranged from 1.6% to 18%.

## Discussion

In most of the FDA oncology drug approvals that required a PMR or PMC it was evident from the FDA reviews that diversity within the clinical study was lacking. To address the low enrollment of a diverse population in clinical studies, FDA issued PMRs and PMCs to further characterize the drug in underrepresented populations in the post–marketing setting. These PMRs and PMCs issued by the FDA are designed to further characterize the safety and efficacy of oncology drugs in a diverse population.

Select drug approvals that had a diversity PMR or PMC issued are described below.

Although sotorasib’s pivotal study had about 18% racial and ethnic minorities in the clinical trial, this is somewhat misleading as a total of 15% of clinical study participants were Asian, and in general the KRAS G12C mutation in NSCLC patients is more common in the White and Black patient population than in Asian patients. Only 1.6% of patients in the pivotal study were Black, which likely led to the FDA issuing a PMC to address the lack of diversity in the trial and to further characterize the safety and efficacy of sotorasib in Black patients.^11^

Another example of a drug approval where the FDA issued a PMC due to an inadequate number of racial and ethnic minority patients is amivantamab-vmjw. Most patients enrolled in the pivotal study for amivantamab-vmjw were White, and only 2.3% of patients were Black. The Sponsor agreed to conduct a study with a sufficient representation of Black patients as it was identified that about 9% of patients with NSCLC with EGFR exon 20 insertion mutations were Black.^12^ Further guidance from the agency on what would constitute a sufficient population to ensure representation (depending on the prevalence of the disease in the US and in different minority populations) would be helpful. In addition, guidance on whether a clinical study, a registry, or real–world evidence from observational studies derived from electronic health records would also be helpful for the drug development community.

Lastly, FDA issued a diversity PMR for melphalan flufenamide due to the differences in safety between White and Black patients in the clinical study. In melphalan flufenamide’s pivotal study, 6% were Black, and the FDA review stated that the data may have demonstrated a potential for a higher rate of dose modifications for hematologic toxicities in racial and ethnic minorities, including Black patients. FDA required the Sponsor to enroll more racial and ethnic minority patients including Black patients to determine if the finding in the review was valid.^13^

The PMRs or PMCs issued by FDA’s Oncology Divisions reflect a need for cancer clinical to enroll a more diverse patient population to reflect the population most likely to take the approved drug. As a result of the lower participation of racial and ethnic minority patients enrolling in cancer clinical trials, the importance to patients and the attention the FDA has put on this topic, Sponsors are making more of a concerted effort to enroll a diverse patient population into clinical trials.^14^ However, it is not without challenges and obstacles.

Enrolling a more diverse patient population is important in cancer clinical trials as it gives a more realistic snapshot of the patients who will be taking the investigational drug to help inform safety and effectiveness. in addition, a diverse patient population may help improve health equity by providing access to investigational drugs that might not be available to them. Although not a focus of this paper diversity in cancer clinical trials also includes older adults as there is a large unmet need in this population. For the vast majority of cancer, clinical trials patients 75 years and older are rarely included or enrollment is low. Due to this disparity and the importance of including older adults FDA issued a Guidance in March 2022 to address and assist sponsors to enroll an adequate representation of this patient population.^15^ In addition to diversity PMRs and PMCs issued by the FDA as related to race and ethnicity, FDA has issued several PMRs and PMCs related to age to urge Sponsors to address this disparity.

However, there are many challenges to enrolling a sufficient population of racial and ethnic minority patients in cancer clinical trials. First, there is an inherent distrust of the medical system due to historical events and abuses that have occurred. In addition, there are issues with health disparities, access and the cost of participating in cancer clinical trials. Lastly, a patient’s lived experience varies based on many factors such as race, ethnicity, geographic location, and education. The issues aforementioned are broad societal issues and cannot be solved by Sponsors alone; however some ways in which Sponsors can aid in the enrollment of racial and ethnic minority patients in cancer clinical trials are to consider selecting sites with higher concentrations of diverse groups, use diverse investigators within cancer clinical trials, and increase patient engagement and education around cancer clinical trials to help with recruitment and retention efforts. Communicating the importance and necessity of enrolling a diverse patient population in trial sites for a trial to be reflective of the US population would also be critical in increasing enrollment of racial and ethnic minorities. In addition, Sponsors could incorporate diversity plans into their cancer clinical trials. The diversity plan may include information regarding the prevalence of the disease in racial and minority groups, expected geographic locations of the studies, and strategies on how to enroll a diverse patient population in clinical trials.^16^

## Conclusion

The number of diversity PMRs and PMCs issued by the FDA’s Oncology Office reflects a renewed emphasis on studying racial and ethnic minority groups during the lifecycle of clinical development to address the lack of diversity in cancer clinical trials. Although there are many challenges and barriers to overcome in increasing enrollment of a diverse patient population in cancer clinical trials, many stakeholders are aware of the importance and necessity of enrollment and are starting to implement strategies to conduct cancer clinical trials that are more reflective of the US population.
